# A Pilot Study Examining Access to and Satisfaction with Maternal Mental Health and Substance Use Disorder Treatment via Telemedicine

**DOI:** 10.1089/tmr.2021.0041

**Published:** 2022-01-11

**Authors:** Constance Guille, Emily Johnson, Edie Douglas, Rubin Aujla, Lisa Boyars, Ryan Kruis, Rebecca Beeks, Kathryn King, Dee Ford, Katherine Sterba

**Affiliations:** ^1^ Department of Psychiatry and Behavioral Sciences and Charleston, South Carolina, USA.; ^2^ Department of Obstetrics and Gynecology; Charleston, South Carolina, USA.; ^3^ Department of College of Nursing; Charleston, South Carolina, USA.; ^4^ Department of Center for Telehealth; Departments of Charleston, South Carolina, USA.; ^5^ Department of Pediatrics, Charleston, South Carolina, USA.; ^6^ Department of Pulmonary and Critical Care, and Charleston, South Carolina, USA.; ^7^ Department of Public Health Sciences; Medical University of South Carolina, Charleston, South Carolina, USA.

**Keywords:** satisfaction, access, telemedicine, perinatal mental health and substance use disorders, pregnancy, postpartum

## Abstract

**Background:** Mental health (MH) and substance use disorders (SUDs) are common during pregnancy and the postpartum year, and have a significant impact on maternal and child health. Most women do not receive treatment for these conditions due to barriers to care. Increasing access to these services via telemedicine is one potential solution to overcoming barriers, but it is unknown if this type of service is acceptable to women. The purpose of this study is to evaluate patient satisfaction with, and accessibility to, a maternal MH and SUD telemedicine service delivered to obstetric practices.

**Methods:** The Telemedicine Satisfaction Questionnaire and the Questionnaire for Assessing Patient Satisfaction with Video Consultation were collected via online surveys. Responses were scored on a 5-point Likert scale, ranging from strongly disagree (1) to strongly agree (5). Paired *t*-tests were used to compare round trip travel time and distance between participants home and specialty clinic at an academic medical center versus their local obstetrics clinic where they received telemedicine services.

**Results:** A total of 91.42% (32/35) of women agreed to take part in the study, and 43.75% (14/32) of women were living in a rural community. Patients reported high levels of satisfaction with the following: overall quality of care (mean [*M*] 4.66 [standard deviation, SD, 0.67]); similarity to face-to-face are (*M* 4.69 [SD 0.63]); and access to care (*M* 4.47 [SD 0.81]). Compared with in-person care at an academic medical center, women receiving care via telemedicine spent significantly less time (67.44 minutes vs. 256.31 minutes, *p* < 0.001) and distance (50.33 miles vs. 236.06 miles, *p* < 0.001) traveling round trip.

**Conclusions:** Women receiving MH and SUD treatment via telemedicine within their obstetrician's office report high levels of satisfaction and increased access to care with this modality of treatment delivery. Telemedicine may provide one solution to removing barriers to care and mitigating the maternal and child risks associated with of untreated MH and SUDs.

## Background

Perinatal mental health (MH) and substance use disorders (SUDs) are recognized as a major public health problem, with 20–30% of women experiencing MH or substance use issues during pregnancy or the year postpartum.^1–5^ Unfortunately, less than 50% of perinatal women with a mental illness are identified in clinical settings.^6^ Among those identified, less than 20% receive MH or SUD treatment, fewer than 10% receive adequate treatment, and less than 5% achieve remission.^6^

There are myriad barriers to MH and SUD care for perinatal women, including insufficient screening and identification of MH and substance use problems.^7,8^ When problems are identified, however, the number of providers capable of, or available to care for these women is insufficient. The average wait times for psychiatric appointments are too long to be of use during the perinatal period.^9^ Furthermore, women often refuse MH treatment or substance use care in psychiatric settings due to the stigma associated with mental illness and substance use.^10,11^

The critical shortage of perinatal psychiatrists and lack of MH and SUD treatment is concerning given that untreated perinatal depression alone is associated with a 1.5-fold increased risk for preterm birth, a 4-fold increased risk of behavioral problems in children at ages 3–4 years; a 2-fold increased risk of poor academic performance in middle school, and a 7-fold increased risk of depression at age 18.^12^ Suicide accounts for 20% of postpartum deaths,^13,14^ and in 2014 over 41.4% of maternal deaths were related to substance use.^15^ Treatment of perinatal MH and SUDs, however, is associated with reduced maternal morbidity and mortality^14^ and improvement in childhood behavioral problems,^12^ but access to specialty care is limited particularly for those living in rural areas.^16,17^

Delivering services to perinatal women in obstetric practices via telemedicine is one potential solution to increasing access to care for women with MH and SUDs. It is unknown, however, if the delivery of these services via telemedicine increases the accessibility of these services and/or if this mode of delivery is acceptable to women. The objectives of this study are to evaluate patient satisfaction with maternal MH and SUD treatment delivered in local obstetric practices via telemedicine and identify accessibility benefits to patients, particularly those living in rural areas.

## Methods

### Recruitment and data collection

Women aged 18–45 years receiving maternal MH and SUD treatment services (defined as at least one appointment with a perinatal psychiatrist) via telemedicine at one of the five obstetric practices were contacted between September 13, 2019, and October 29, 2019, and were invited to take part in the study. Women were not invited to take part in the study if they were currently experiencing psychotic symptoms or unable to provide informed consent. Potential participants were contacted by their MH provider and asked if they would be willing to complete a brief confidential online survey as part of a research study. All women had received synchronous psychiatric services, including evaluation, evidence-based therapy, and/or medication management, from one of the four perinatal psychiatrists via Vidyo and/or Jabber telemedicine software.

Patients interested in hearing more about the study agreed to have a research coordinator contact them by phone to review the study's Statement of Research. Participants verbally agreeing to take part in the study were sent a link to an online survey via e-mail. Participant remuneration included a $20 gift card. The Medical University of South Carolina Institutional Review Board approved this study and granted a waiver of written informed consent (protocol no. 00086783).

### Measures

Demographic characteristics (age, race, and ethnicity) were self-reported by participants. MH and SUD diagnoses, defined by the *Diagnostic and Statistical Manual of Mental Disorders, 5th Edition*,^18^ were extracted from the electronic medical record. Rurality status (yes or no) based on a patient's home address was defined by the Health Resources and Services Administration (HRSA) Rural Analyzer.^19^ Travel time was defined as the average number of minutes and miles to the local obstetrics practice where telemedicine services were delivered and the academic medical site where the participant would have received care if telemedicine were not available. Travel time and distance were calculated using Google Maps with participant home and clinic addresses.

Satisfaction with overall quality of care (8 items) and similarity with face-to-face care (5 items) were assessed using two subscales from the previously validated Telemedicine Satisfaction Questionnaire.^20^ Cronbach's alphas, a measure of reliability or internal consistency in the current study sample, were good (e.g., 0.86 and 0.75, respectively). Satisfaction with access to care (4 items) was assessed using a subscale from the Questionnaire for Assessing Patient Satisfaction with Video Consultation.^21^ The Cronbach's alpha in the current study sample was 0.80. Finally, satisfaction with MH care delivery was assessed using seven investigator-developed items, which had not been previously validated, to capture perceived benefits of MH care in the telehealth setting (Table 2). Responses to all subscales were indicated on a 5-point Likert scale ranging from 1 = strongly disagree to 5 = strongly agree.

### Data analysis

Descriptive statistics were used to describe patient demographics, the *Diagnostic and Statistical Manual of Mental Disorders, 5th Edition* diagnosis, and rurality characteristics. Paired *t*-tests were used to evaluate differences between travel time (minutes) and distance (miles) between in-person care at an academic medical center versus care at a local obstetric telemedicine site. These paired *t*-tests were completed in the overall group and also in those living in rural and nonrural settings separately. To counteract the problem of multiple comparisons, a Bonferroni correction was applied due to multiple *t*-tests (*n* = 4) and our modest sample size. Thus, findings were considered statistically significant at an alpha set at *p* ≤ 0.0125 (0.05/4). Descriptive statistics (mean [*M*] and standard deviation [SD]) were used to characterize patient satisfaction with telemedicine services. Multi-item scales were averaged for each of the satisfaction domains (overall quality of care, similarity with face-to-face care, and access to care), and averages were presented for each individual satisfaction item and MH care items, with higher scores reflecting higher satisfaction. SAS 9.4 was utilized for all analyses.

## Results

A total of 35 women receiving care at one of five telemedicine sites were invited to take part in the survey and 32 completed the online survey (91.43% participation rate). The majority of women were between 25 and 34 years of age (46.87% [15/32]), 75% (24/32) self-identified as white and 43.75% (14/32) of patients were living in rural settings (Table 1). The most common MH diagnoses were mood (56.25% [18/32]) and anxiety (52.13% [17/32]) disorders. Opioid use disorders were the most common SUDs (40.62% [13/32]) (Table 1).

**Table 1.** Participant Demographics (*n* = 32)

Characteristic	*n* (%)
Age	
18–24 years	8 (25.00)
25–34 years	15 (46.87)
35–44 years	9 (28.13)
Race	
White	24 (75.00)
Black	5 (15.63)
Unknown/other	3 (9.38)
Ethnicity	
Hispanic	2 (6.25)
Not Hispanic	28 (87.50)
Unknown	2 (6.25)
DSM-V psychiatric diagnosis, *n* (%)	
Mood disorder (all types)	18 (56.25)
Anxiety disorder (all types)	17 (52.13)
Post-traumatic stress disorder	9 (28.13)
Attention-deficit disorder	5 (15.63)
DSM-V substance use disorder, *n* (%)	
Opioid	13 (40.62)
Cannabis	5 (15.62)
Alcohol	1 (3.13)
Sedative/hypnotics	1 (3.13)
Cocaine	1 (3.13)
Amphetamine	1 (3.13)
Rurality, *n* (%)	
Living in rural setting	14 (43.75)
Round trip travel time, telemedicine site, *M* (range)	67.44 (16.00–178.00) minutes
	50.33 (7.20–157.40) miles
Round trip drive time, specialist site, *M* (range)	256.31 (160.00–346.00) minutes
	236.06 (127.80–338.00) miles

DSM-V, *Diagnostic and Statistical Manual of Mental Disorders, 5th Edition*.

The average round trip distance for travel from home to the local telemedicine clinic was significantly less than the average round trip distance from home to the academic medical center providing specialty services (*M* 50.33 miles [SD 38.58] vs. *M* 236.06 miles [SD 64.05]; *t*(31) = −16.52, *p* < 0.001). Similarly, average round trip time for travel from home to the local telemedicine clinic was significantly less than the average round trip time from home to the academic medical center providing specialty services (*M* 67.44 minutes [SD 42.55] vs. *M* 256.31 minutes [SD 45.99]; *t*(31) = −22.93, <0.001).

On average, patients traveled 185.73 fewer miles and 188.88 fewer minutes round trip to the local telehealth clinic, compared with distance and travel time to the specialty clinic, respectively (Table 1). These differences in distance and time were significant for both those living in rural (*n* = 14) and nonrural (*n* = 18) settings (*p'*s < 0.001).

Patients reported high levels of satisfaction with the maternal mental telehealth services across all satisfaction domains. High satisfaction scores were reported for similarity to face-to-face care (*M* 4.69 [SD 0.63]), overall quality of care (*M* 4.66 [SD 0.67]), and access to care (*M* 4.47 [SD 0.81]) domains (Table 2). Similarly, high satisfaction scores were reported for MH care access and quality of care (Table 2).

**Table 2.** Participant Satisfaction (*n* = 32)

Telemedicine satisfaction domain^a^	Mean	Standard deviation
Overall quality of care^20^	4.66	0.67
I do not need assistance while using the system.	4.59	0.61
I think the health care provided via telemedicine is consistent.	4.66	0.79
I obtain better access to health care services by use of telemedicine.	4.53	0.67
I do receive adequate attention.	4.53	1.08
Telemedicine provides for my health care need.	4.75	0.51
I find telemedicine an acceptable way to receive health care services.	4.72	0.52
I will use telemedicine services again.	4.66	0.60
Overall, I am satisfied with the quality of service being provided via telemedicine.	4.81	0.40
Similarity to face-to-face care^20^	4.69	0.63
I can easily talk to my health care provider.	4.96	0.54
I can hear my health care provider clearly.	4.69	0.64
My health care provider is able to understand my health care condition.	4.75	0.44
I can see my health care provider as if we met in person.	4.68	0.54
Telemedicine saves me time traveling to hospital or a specialist clinic.	4.63	0.91
Access to care^20^	4.47	0.81
Telehealth enables me to save money and time.	4.47	0.98
Telemedicine improves my access to specialist care.	4.69	0.54
I believe that treatment via telemedicine helps reduce my MH or substance use symptoms.	4.38	0.79
I prefer to have my next consultation via telemedicine.	4.34	0.87
MH care (investigator-developed)		
It is easier to access MH care through telemedicine, compared with in-person MH care.	4.38	0.91
I had to wait a long time to get an appointment with a telemedicine MH provider.	1.84	1.08
My geographic location makes it hard to access telemedicine MH treatment.	1.94	1.18
It is difficult for me to find transportation to my telemedicine MH appointments.	1.87	1.23
My telemedicine MH provider and my other health care providers coordinate and collaborate about my care.	4.40	0.90
I can trust my telemedicine MH provider.	4.75	0.80
I feel that treatment decisions are made in partnership with my telemedicine MH provider.	4.47	1.08

^a^ Response set for all scales from 1 (strongly disagree) to 5 (strongly agree).

MH, mental health.

## Discussion

Pregnant and postpartum women receiving MH and/or SUD care via telemedicine within their local obstetrician's office report high levels of satisfaction with their overall care. In addition, they indicate that telemedicine services are similar to face-to-face care and improve their overall access to MH and SUD treatment. In fact, the majority of participants agreed that it is easier to access services via telemedicine, compared with in-person care.

Overall, these findings suggest that women living in both rural (44% of sample) and nonrural areas (56% of sample) perceive that telemedicine service improves their access and ease of access to MH and SUD treatment. These findings are important given that the vast majority of women who screen positive for perinatal MH problems do not follow through with referrals to treatment^8^ and less than 20% of pregnant women with SUDs receive treatment.^4^

Participants' perceptions that MH treatment via telemedicine improves overall access and ease of access to services may potentially be due to the fact that this service modality overcomes many common barriers to MH and SUD treatment for perinatal women.^8^ For example, participants' responses suggest that telemedicine services overcome barriers such as long wait times for MH appointments, lack of transportation, and geographic location or distance to clinics.

These findings are further supported by our evaluation of both travel time and distance to the local obstetrician's office compared with a specialty provider at an academic center. On average, participants traveled 185.73 fewer miles and 188.88 fewer minutes (∼3 fewer hours) round trip to the local telehealth clinic, compared with distance and travel time to the specialty clinic, respectively.

Our findings demonstrate that telemedicine services delivered locally within an obstetrician's office, compared with an academic setting, significantly reduced both distance and travel time to appointments, and these differences held for participants living in both rural and nonrural settings.

Participants' perceptions that access to MH and SUD services is easier with telemedicine compared with in-person care are possibly due to the colocation of telemedicine services with obstetrics care as opposed to treatment outside of the obstetric setting. In addition to the ease and convenience of colocation of services, participants also agreed that there was coordination and collaboration of care between specialists. These findings are encouraging given the known benefits of integrated behavioral health care for perinatal women with MH^22,23^ and SUDs,^4,24,25^ and suggest that collaboration between specialists can be achieved, from the patient's perspective, via telemedicine.

Beyond the perception that telemedicine increases access to care and enhances important elements of integrated care, participants also agreed that telemedicine services were similar to “face-to-face” treatment. Importantly, participants indicated that they experienced feelings of trust and partnership in health care decisions with their provider. These findings suggest that core elements of the patient/physician relationship such as trust and shared-decision can be achieved via telemedicine.^26,27^

This study is not without limitation. First, participants in this study were those who were currently receiving MH and SUD services via telemedicine. It would be important to gather feedback from women who declined telemedicine services and understand the reasons why they did not engage in this treatment delivery modality. Second, while the measures used in this study reflect current validated telemedicine satisfaction scales, there are very few validated scales available to assess patient satisfaction with telemedicine, and qualitative research could offer a more in-depth understanding of care experiences and improve the validity of findings.

Third, while our findings demonstrate that participants traveled less for telemedicine appointments compared with if they had received care in-person at an academic center, it is possible that women could have received in-person MH services locally. Lastly, this study was modest in size and cross-sectional in design. Future larger longitudinal studies that capture more in-depth evaluations of satisfaction with and potential benefits of telemedicine for perinatal women are warranted, including larger studies with sufficient power to examine results by subgroups, including age, race, and/or diagnosis, as well as more in-depth evaluation of the potential benefits of virtual colocation, coordination, and collaboration of care using telemedicine.

## Conclusions

Perinatal MH and SUDs are common and carry significant morbidity and mortality for women and their children. Unfortunately, however, the majority of women will not receive any or adequate treatment due to barriers-to-care. The delivery of maternal MH and substance use services via telemedicine to obstetric offices appears to increase the accessibility and availability of these services and this mode of delivery is acceptable, and in some case more favorable to women, compared with in-person services.

Furthermore, pairing both prenatal care and MH and substance use services in one location is likely more convenient for women and potentially increases communication and coordination of care between obstetric and MH providers. Telemedicine, therefore, may be one potential solution to removing barriers to MH and SUD care and increasing access to needed services for peripartum women living in rural and nonrural areas. Overcoming common barriers to care and creating easier access to services for peripartum women are likely one way to increase engagement in treatment and reduce the significant morbidity and mortality associated with perinatal MH and substance use.

## Abbreviations Used

HRSA: Health Resources and Services Administration
MH: mental health
SD: standard deviation
SUDs: substance use disorders
